# Contrast-Enhanced Ultrasound (CEUS) and Ultra-Microangiography (UMA) in Critically Ill Children with Acute Kidney Injury

**DOI:** 10.3390/children11101205

**Published:** 2024-09-30

**Authors:** Nace Ogorevc, Peter Slak, Stevan Nikšić, Gregor Novljan, Petja Fister, Domen Plut

**Affiliations:** 1 Clinical Radiology Institute, University Medical Centre Ljubljana, 1000 Ljubljana, Slovenia; peter.slak@kclj.si (P.S.); domen.plut@kclj.si (D.P.); 2 Faculty of Medicine, University of Ljubljana, 1000 Ljubljana, Slovenia; 3 Pediatric Nephrology Department, Children’s Hospital, University Medical Centre Ljubljana, 1000 Ljubljana, Slovenia; 4 Department of Pediatric Intensive Care, Children’s Hospital, University Medical Centre Ljubljana, 1000 Ljubljana, Slovenia

**Keywords:** acute kidney injury, contrast-enhanced ultrasound, ultra-microangiography

## Abstract

Acute kidney injury (AKI) is an acute condition of impaired kidney function with decreased glomerular filtration rate, which results in dysregulation in volume, electrolyte, and acid–base equilibrium. AKI can be a life-threatening condition and can also lead to chronic kidney disease. It is important to diagnose AKI early in the course of the disease or to predict its development, as this can influence therapeutic decisions, outcome, and, consequently, the prognosis. In clinical practice, an elevated serum creatinine concentration remains the most common laboratory indicator for diagnosing AKI. However, due to the delay in its rise, creatinine levels are often insensitive and inaccurate for early diagnosis. Novel biomarkers of kidney tubular injury and the renal angina index have shown promise in predicting AKI earlier and more accurately. Contrast-enhanced ultrasonography (CEUS) and ultra-microangiography (UMA) are radiological methods that can quantify renal microperfusion and may be able to predict the development of AKI. They have not yet been used for quantifying renal perfusion in children with risk factors for developing AKI. Further research is needed to compare these sonographic techniques with the renal angina index and emerging kidney injury biomarkers for predicting acute kidney injury (AKI) in both children and adults.

## 1. Introduction

Acute kidney injury (AKI) is an acute condition of impaired kidney function with decreased glomerular filtration rate and resulting dysregulation in volume, electrolyte, and acid–base equilibrium [1]. Diagnosing AKI early in the course of the disease is important, as this can influence therapeutic decisions (fluid therapy, earlier initiation of renal replacement therapy, discontinuation of nephrotoxic drugs) and, consequently, improve the outcome and long-term prognosis [2]. Since serum creatinine concentrations are frequently a late and imprecise indicator of AKI, research has aimed to discover biomarkers that can reliably predict or detect AKI in its early stages. New laboratory biomarkers, including neutrophil gelatinase-associated lipocalin, kidney injury molecule-1, interleukin 18, fibroblast growth factor 23, insulin growth factor-binding protein 7, cystatin C, urinary calprotectin, urine retinol-binding protein 4, urinary liver-type fatty acid-binding protein, clusterin, and tissue inhibitor of metalloproteinases 2, appear to be promising for both diagnosing and forecasting AKI in children but are not often routinely used [3,4,5,6]. The novel biomarkers have been associated with a faster response time and a higher sensitivity early in the disease course for AKI detection than serum creatinine [7,8,9,10,11,12,13,14,15,16,17,18]. The Renal Angina Index (RAI) is a prognostic tool designed for use upon admission to a pediatric intensive care unit (PICU) to predict the likelihood of progressing to more severe stages of AKI. The RAI integrates risk factors for AKI development (i.e., PICU admission, solid organ or stem cell transplantation, and ventilation with inotropy) and clinical indicators of kidney dysfunction (i.e., fluid overload and the change in serum creatinine clearance). An RAI score is calculated by multiplying the values of the risk factors by those of the clinical signs of kidney impairment. Scores range from 1 to 40, with a score of 8 or higher suggesting the fulfillment of renal angina [19]. The RAI has proven to be a more accurate and superior predictor of higher stages of AKI in critically ill children and young adults compared to creatinine concentrations [20]. Despite its potential, the RAI has notable drawbacks: limited sensitivity (85%) and specificity (79%) for AKI prediction, inconsistent applicability beyond pediatric populations, limited influence on guiding treatment, and exclusion of some AKI risk factors [21]. Unfortunately, macrohemodynamic (cardiac index, mean arterial blood pressure) and microhemodynamic measurements (resistance index and flow in the renal artery) have not been proven to be sensitive and specific enough for the early prediction of AKI development [22]. Novel sonographic techniques, such as contrast-enhanced ultrasound (CEUS) and ultra-microangiography (UMA), offer detailed visualization and quantification of renal microvasculature, surpassing the capabilities of traditional grayscale and Doppler ultrasound. These methods could contribute to the earlier diagnosis/prediction of prerenal AKI. Several studies in the adult population show the benefit of CEUS [22,23,24,25,26,27], while UMA is yet to be investigated for this purpose [28]. In this article, we aim to review the pathophysiology and definition of AKI, technological aspects of CEUS and UMA, and previous research to determine their potential for the earlier diagnosis/prediction of prerenal AKI in children.

## 2. Acute Kidney Injury: Definition, Epidemiology, Etiology and Risk Factors

In pediatric AKI, severity is typically classified into three stages using Kidney Disease: Improving Global Outcomes (KDIGO) criteria based on serum creatinine levels and/or urine output [29,30,31,32]. Baseline serum creatinine for this classification is determined by age and gender norms [33,34]. AKI is common in newborns and infants in intensive care units and is associated with increased morbidity and mortality [35]. In the last decades, AKI rates in children have considerably grown [36]. This is due to the more complex and advanced medical management, aggressive treatment protocols, demanding surgical procedures, higher incidence of chronic conditions, and more premature surviving children. Rates of AKI in hospitalized critically ill children reach 55% [37,38,39], with moderate to severe AKI representing 10–14% [40]. Newborns and infants are more susceptible to developing AKI due to immature kidney function and an immature immune system (leading to more frequent infections and sepsis), reduced ability to concentrate urine, and a higher surface-area-to-weight ratio, which predisposes them to dehydration. Additionally, since most nephrogenesis occurs in the third trimester of pregnancy, preterm newborns and growth-restricted neonates, both groups with a lower number of nephrons at birth, have a higher risk of developing AKI [41]. The increased resistance of renal vasculature, which predisposes newborns and infants to renal parenchymal ischemia upon a triggering factor, gradually decreases in the first two years of life. Consequently, renal blood flow increases and glomerular filtration reaches adult levels [23].

Newborns and infants are at increased risk for developing AKI, particularly those born prematurely and with growth restriction, those with congenital heart disease needing cardiac surgery, those with hypoxic–ischemic encephalopathy, necrotizing enterocolitis, and those for whom nephrotoxic drugs are indicated (most commonly gentamicin, vancomycin, NSAIDs, and acyclovir) [2]. Other risk factors in children include sepsis, shock of any etiology, hypoxemia, malignancies, use of mechanical ventilation, and vasopressor support [42,43]. Approximately 11% of pediatric patients admitted to the hospital for viral bronchiolitis outside of a pediatric intensive care unit (PICU) setting experience acute kidney injury, which is most often mild in severity [44]. Acute kidney injury affects 14.6% of children hospitalized with febrile urinary tract infections (fUTI), and its prevalence doubles in those with congenital kidney and urinary tract anomalies during fUTI episodes [45]. Around 25% of patients hospitalized with acute gastroenteritis may develop acute kidney injury, typically mild in severity [46].

AKI in children is etiologically classified as prerenal, renal, or postrenal. Most often (in 65–75% of cases), the cause of AKI is prerenal: due to hypovolemia or reduced effective circulatory volume. In the intensive care units, the cause of AKI is most likely multifactorial [47,48]. Between 1978 and 2014, eight longitudinal studies revealed a significant prevalence of chronic kidney disease (CKD) among newborns who experienced AKI, with incidence rates being widespread, ranging up to 66%. The risk was higher for those who had more severe stages of AKI and multiple episodes [49]. According to the expert group, all newborns who have recovered from AKI (especially at higher stages) should be followed up. The KDIGO guidelines recommend assessing the development of chronic kidney disease three months after an AKI episode by measuring serum creatinine concentration, albuminuria, and arterial blood pressure (hypertension) [35].

## 3. Limitations of Established Methods for Diagnosing AKI

Serum creatinine concentration is highly specific for detecting AKI, but it lacks sensitivity for early diagnosis because its concentration rises only 48–72 h after kidney injury, regardless of the etiology. By the time creatinine concentration rises, which occurs only after a reduction in glomerular filtration by more than 50%, irreversible kidney damage has already occurred [50]. Serum creatinine concentration often provides imprecise results, as it indicates glomerular filtration rate (GFR) in individuals with stable kidney function and does not accurately represent GFR in patients with fluctuating or rapidly changing kidney function. Additionally, serum creatinine concentration depends on many factors, such as age, sex, muscle mass, hydration, and nutritional status [2]. Creatinine is also eliminated through dialysis, making it impossible to evaluate kidney function using serum creatinine concentrations after dialysis has begun [51,52]. Furthermore, an increased creatinine concentration reflects impaired kidney function rather than the kidney injury itself [50]. A frequently missed issue is that maternal serum creatinine can affect a newborn’s creatinine concentration at birth since creatinine crosses the placenta. In full-term newborns, its concentration typically normalizes within a week, while preterm newborns with less mature kidneys experience higher concentrations that decrease more gradually over several weeks [29,30,31]. The problem with using urine output for diagnosing AKI in the pediatric intensive care unit according to the KDIGO criteria is that it is influenced by the amount of fluids administered and the use of diuretics [53]. When urine output criteria fail to detect or classify polyuric AKI, reliance must shift solely to the serum creatinine criterion to assess and diagnose the condition. In children with type 1 diabetes and diabetic ketoacidosis, the prevalence of acute kidney injury (AKI) reaches up to 65%. However, the frequent occurrence of polyuria and polydipsia in this group makes the urine output criterion from the KDIGO classification less reliable for diagnosing AKI. Only 15% of children with AKI at the onset of type 1 diabetes fulfilled the KDIGO urine output criteria [54,55]. In diabetic ketoacidosis, dehydration leads to concentrated blood components, including creatinine, which can falsely elevate its levels even without real kidney damage. During treatment, aggressive rehydration with fluids dilutes creatinine, potentially lowering its concentration even if kidney function is still impaired [56].

## 4. Sonographic Findings in AKI

Sonographic findings in AKI, aside from postrenal causes inducing hydronephrosis, are often insensitive and cannot distinguish between different causes of AKI (acute tubular necrosis, tubulointerstitial nephritis, glomerulonephritis, pyelonephritis, etc.). On ultrasound, the kidneys in AKI are usually of normal or increased size and hyperechogenic. The echogenicity of the cortex should normally never be greater than that of the normal liver parenchyma. However, in prerenal AKI, the cortex is typically of normal echogenicity, which reduces the sensitivity of detecting AKI [57]. Additionally, the kidneys in AKI due to hypotension may appear hypoechoic due to edema caused by ischemia (acute tubular necrosis) [58]. Another finding that may be present in AKI is a thin, hypoechoic rim in the perirenal space representing edema/fluid [59].

## 5. Contrast-Enhanced Ultrasound (CEUS) in AKI

The use of sonographic methods with contrast agents (contrast-enhanced ultrasound—CEUS)—microbubbles, has seen a surge in clinical and research applications in recent years due to its noninvasiveness, diagnostic value for multiple organ systems, and lack of serious side effects [60]. The Food and Drug Administration (FDA), responsible for drug safety in the USA, approved the use of an ultrasound contrast agent for intravenous use in children in 2016, specifically for assessing focal liver lesions. However, since the ultrasound contrast agent distributes uniformly throughout the body regardless of the indication, its off-label use has significantly expanded to other indications. The European Federation of Societies for Ultrasound in Medicine and Biology (EFSUMB) has recommended its intravenous use in children for many years [61]. CEUS does not require any special preparation, sedation, or anesthesia. The method is safe, accessible, and quick; it can be performed at the bedside of a newborn in the intensive care unit and repeated as needed. It does, however, require a trained operator and specialized equipment. In a retrospective study conducted on children (in 29 European centers), 948 CEUS examinations were performed for various indications, with reports of five mild side effects [62]. Microbubbles have a gas-filled core surrounded by a shell composed of phospholipids or albumin. They are the size of red blood cells, allowing them to pass through capillaries. In the absence of bleeding, they remain strictly intravascular. They can resonate with the frequency of ultrasound waves, enabling their detection and, thus, the visualization of the microvasculature. Microbubbles are quickly eliminated through the lungs and the reticuloendothelial system, so it is unnecessary to check kidney and liver function before use [63]. Currently, two primary methods are utilized to estimate tissue microvascular perfusion using CEUS: bolus kinetics and flash-replenishment kinetics. In the bolus technique, a bolus of ultrasound contrast agent is injected intravenously, and a time-acoustic intensity curve is recorded. Conversely, flash-replenishment kinetics involves the reappearance of contrast enhancement following the destruction of microbubbles by a high-intensity flash during continuous intravenous infusion of the contrast agents [64].

**Figure 1:**
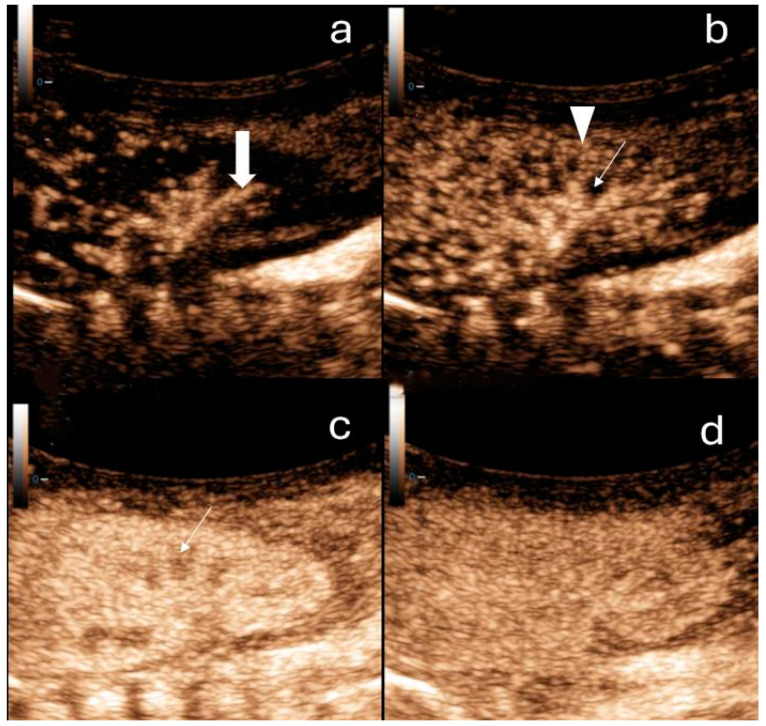
Contrast-enhanced ultrasound of the right kidney in a newborn. (**a**) Arterial phase: a few seconds after contrast administration interlobar arteries of the kidney can be observed (thick arrow). (**b**) Cortical phase: a few seconds after the arterial phase the contrast distributes within the renal cortex (arrowheads). The medulla (arrow) is not enhancing. (**c**) Parenchymal phase: after approximately 20 s, the outer medulla is filled with contrast (arrow). (**d**) In the late phase (2 min after contrast application), the pyramids are fully enhanced.

**Figure 2:**
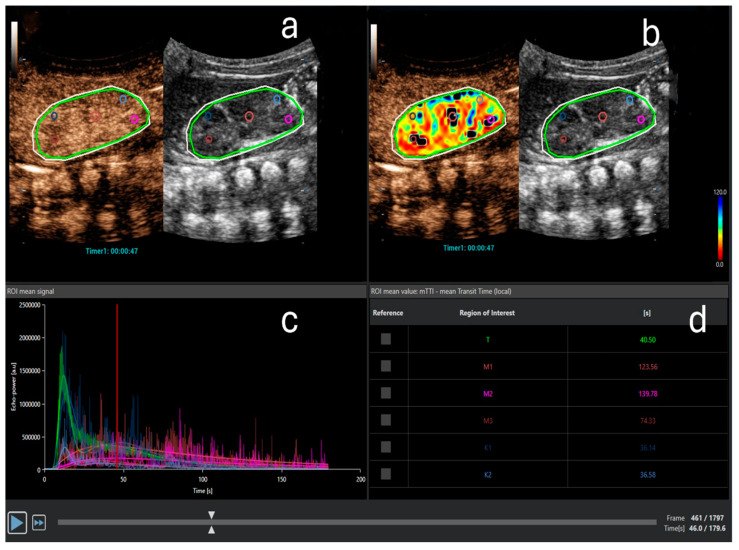
Contrast-enhanced ultrasound quantitative analysis. (**a**) Three regions of interest (ROIs) in the medulla (pink, purple, and red) and two ROIs in the cortex (light and dark blue) are designated. The light green oval ROI represents the entire kidney. The outer white ROI represents the area where analysis is performed. (**b**) Perfusion map for the parameter mean transit time (mTT) with selected ROIs. (**c**) Time-intensity curve (TIC): echo power in arbitrary units on the ordinate and time on the abscissa. Different curves represent the anatomical regions, marked with the same color as the ROIs in (**a**,**b**). Note the typical shape of the curve after bolus-contrast administration. (**d**) The quantitative analysis for mTT in seconds for the entire kidney (t), cortex (K1, K2), and medulla (M1–M3). mTT is the time to 50% of peak signal intensity and is prolonged in the medulla compared to the cortex due to the physiological renal perfusion, where the cortex receives most of the blood volume earlier than the medulla.

One of the current research applications of CEUS is the assessment of renal perfusion before the onset of AKI, at its diagnosis, and during subsequent monitoring. The 3 min dynamic enhancement recordings of the kidney performed with CEUS (Figure 1) are saved and transferred to the quantification software, where specific regions of interest (ROIs) are designated. ROIs can be marked in both the cortex and medulla of the kidney. The software then quantifies renal perfusion from the recording at chosen ROIs and displays results using time–intensity curves (TICs), which provide volumetric, temporal, and other parameters (Figure 2). Volumetric parameters include the maximum signal intensity, which is proportional to the blood volume in the measured area of the kidney, and the area under the curve. The most useful temporal parameters include the time to peak and the time to 50% of peak signal intensity (mean transit time). Other parameters include the slope of the TIC (wash-in and wash-out rate) [26].

The only systematic review and meta-analysis on CEUS in AKI included six studies on adult patients. They found that patients with AKI, most commonly of prerenal origin (typically due to shock, often septic), had significantly reduced renal microcirculatory perfusion (reduced maximum cortical signal intensity), prolonged perfusion times, and a flatter slope of the TIC compared to patients who did not have AKI or did not develop it later during hospitalization [26]. Additionally, CEUS indicators in patients who later developed AKI according to KDIGO criteria were abnormal before the rise in serum creatinine concentration, which could aid in the earlier prediction, diagnosis, and prevention/treatment of AKI [26]. No research has yet been conducted to study the predictive power of CEUS parameters in AKI in the pediatric population. One study has been undertaken in children that investigated the utility of CEUS for assessing renal microperfusion impairment, specifically in children with chronic kidney disease [63]. The study compared the renal cortex and medulla within the same kidney, and the TIC-derived CEUS parameter values showed no significant differences among the three sampled ROIs in both regions. Additionally, when comparing the left and right kidneys, the TIC-derived CEUS parameter values revealed no significant differences between the two sides in either the renal cortices or medullas. These findings confirmed the consistency of CEUS quantitative analysis in pediatric kidneys. The same study demonstrated that as pediatric CKD progresses, the value of parameter A, which reflects the regional blood volume (maximum signal intensity) in the renal cortex, decreases [63]. Research on adult patients also indicates that CEUS measurements can predict the likelihood of developing chronic kidney disease [23]. A study evaluating the repeatability of CEUS to determine renal cortical perfusion showed good intraindividual and excellent interrater repeatability [65]. Another study combined two CEUS parameters, perfusion index and wash-in slope with serum creatinine concentration, for the early diagnosis of AKI in septic adult patients and found a high diagnostic accuracy (area under curve (AUC) 0.943) and sensitivity and specificity of this combination of 94.59% and 81.13%, respectively [24].

## 6. Ultra-Microangiography (UMA)

**Figure 3:**
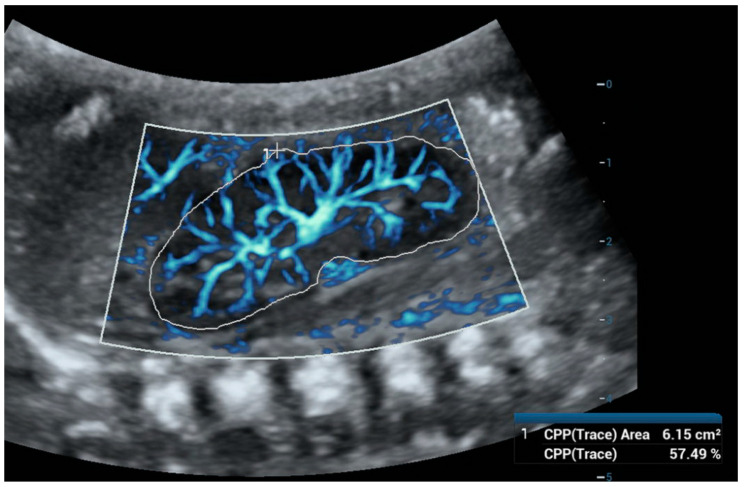
Ultra-microangiography (UMA) of a newborn’s right kidney using a convex abdominal probe. To calculate the Vascular Index (VI), we used the color pixel percentage (CPP)—trace function and outlined the outer borders of the kidney. The VI was 57.49%.

**Figure 4:**
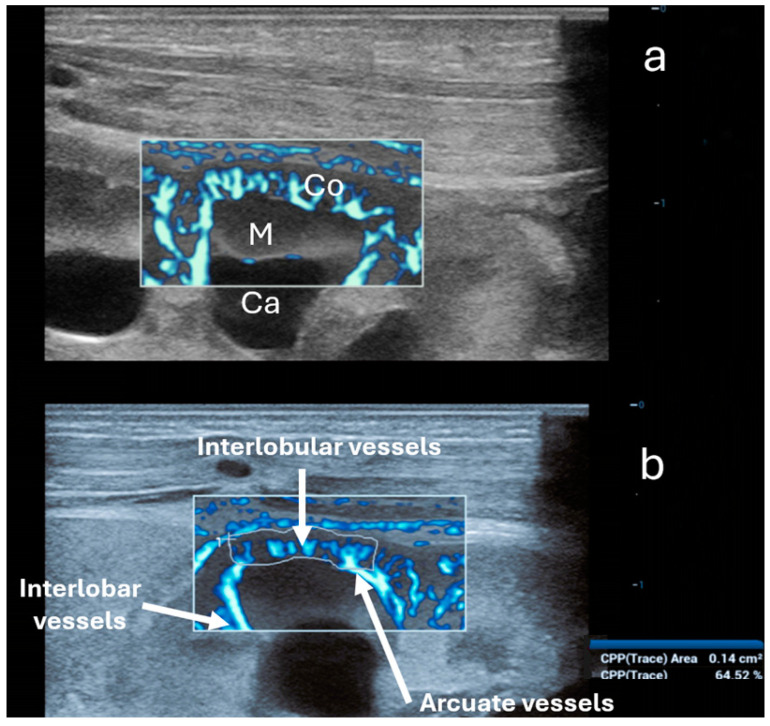
Ultra-microangiography (UMA) of the renal cortex (Co) and a medullary pyramid (M) in the same newborn using a linear 20 MHz probe. Ca—minor calyx. (**a**) Interlobar vessels traverse Bertin’s columns between two adjacent renal pyramids. Interlobar vessels branch into the arcuate vessels. The latter creates an arch above the medullary pyramids, giving off the interlobular vessels that radiate peripherally perpendicular toward the cortex. (**b**) Calculation of the vascular index solely of the cortex (64.52%) using the trace option (below).

Ultra-microangiography (UMA) is a novel sonographic method that measures low-velocity flows in small blood vessels with high resolution, making it more sensitive than color, power, and spectral Doppler. This method is similar in use to the Doppler techniques; however, it is only available in newer US machines. UMA shows a sensitivity of nearly 82% (similar to CEUS) in detecting flow in solid renal lesions, compared to 42% with color Doppler imaging and 47% with power Doppler imaging [66]. Technically, UMA is an advanced Doppler imaging technique, which incorporates a sophisticated wall filtering algorithm that effectively differentiates slow tissue movements from blood flow signals. UMA uses plane waves at a very high frame rate and an intelligent tissue suppression algorithm (adaptive spatiotemporal tissue-rejection filtering) that effectively eliminates tissue interference from raw ultrasound signals, which allows for better detection of low-velocity blood flow [67,68,69]. UMA also enables flow quantification using the Vascular Index (VI), which represents the ratio of points with flow signal to all points in the captured area and could, therefore, potentially contribute to kidney microperfusion measurement (Figure 3 and Figure 4). In kidney assessments, UMA has proven useful for evaluating renal cystic lesions, distinguishing between benign and malignant lesions based on vascularity, and diagnosing renal infarctions and traumatic lacerations [28]. In the literature, we have not found any data on trials evaluating the use of UMA techniques for assessing renal perfusion in AKI. However, we found a study using superb microvascular imaging (SMI), one of the UMA techniques, for the early assessment of chronic renal morphological changes (fibrosis) and grading chronic kidney disease stages in adults. The study demonstrated that the VI was significantly lower in patients with chronic kidney disease compared to healthy individuals. Additionally, it was found that the VI was significantly lower in patients with higher stages of chronic kidney disease, which were associated with more reduced eGFR and higher serum creatinine concentrations. The correlation between lower VI and proteinuria was mild, and the VI was also associated with the degree of kidney fibrosis proven by biopsy [70].

## 7. Conclusions

Currently established methods for diagnosing acute kidney injury in children are not ideal and there is a need for improvement, specifically in more reliable early detection/prediction. In recent years, numerous studies have explored the use of CEUS and UMA in assessing renal pathology. Because of the ability of these methods to quantitatively evaluate renal microvasculature, they may prove to be a reliable tool in predicting prerenal AKI, and further research in this field is warranted. However, these newer techniques require specific equipment and a trained operator; therefore, their use is not yet widespread. Additional studies are warranted to compare sonographic methods with RAI and novel kidney injury biomarkers in predicting AKI in children and adults, as well as to prognosticate the development of chronic kidney disease.

## Data Availability

Not applicable.
